# RAPD-PCR-Based Fingerprinting Method as a Tool for Epidemiological Analysis of *Trueperella pyogenes* Infections

**DOI:** 10.3390/pathogens11050562

**Published:** 2022-05-10

**Authors:** Ilona Stefańska, Ewelina Kwiecień, Małgorzata Górzyńska, Agnieszka Sałamaszyńska-Guz, Magdalena Rzewuska

**Affiliations:** Department of Preclinical Sciences, Institute of Veterinary Medicine, Warsaw University of Life Sciences, Ciszewskiego 8 St., 02-786 Warsaw, Poland; malgorzata_dasiewicz@wp.pl (M.G.); agnieszka_salamaszynska_guz@sggw.edu.pl (A.S.-G.); magdalena_rzewuska@sggw.edu.pl (M.R.)

**Keywords:** genetic diversity, epidemiology, infections, molecular typing, optimization, RAPD-PCR, *Trueperella pyogenes*

## Abstract

In this study, a Random Amplified Polymorphic DNA-Polymerase Chain Reaction (RAPD-PCR) method for genetic typing of *Trueperella pyogenes*, an opportunistic bacterial pathogen, was designed. The method optimization was performed for 37 clinical *T. pyogenes* strains isolated from various infections in different animal species. Optimal conditions for reliable and reproducible DNA fingerprinting were determined according to the modified Taguchi method. The developed method was assessed regarding its typeability, reproducibility, and discriminatory power using the Hunter’s and Gatsons’ index of discrimination. A high degree of genetic diversity was shown between the studied strains, which represented 31 genotypes. The generated RAPD profiles were relatively complex and simultaneously easy to interpret due to the wide size range of amplicons. The discriminatory index of the designed method was sufficiently high; thus, only strains epidemiologically related displayed identical RAPD genotypes. In conclusion, the DNA fingerprinting of *T. pyogenes* by the developed RAPD-PCR method is a reliable typing tool that may allow a better understanding of the epidemiology as well as pathogenesis of infections caused by this pathogen.

## 1. Introduction

*Trueperella pyogenes*, a Gram-positive irregular rod, is an inhabitant of the mucus membranes of the upper respiratory, gastrointestinal, and urogenital tracts of animals. Nevertheless, this bacterium can be opportunistically pathogenic for both livestock and wild animals, causing different purulent infections [1]. *T. pyogenes* infections are especially important in breeding animals, including swine, cattle, and small ruminants, because they lead to serious economic losses [2,3]. Although this opportunistic pathogen has been known for a long time, the pathogenesis and routes of infections it causes are still poorly understood. Moreover, little is known about the sources and dissemination of *T. pyogenes* strains amongst domestic animals as well as wildlife.

The knowledge of genetic diversity and relationships of bacterial strains associated with various infections is crucial for epidemiological investigations and is also a basis for estimation of the pathogenic potential of bacteria. The Pulsed-Field Gel Electrophoresis (PFGE) is regarded as one of the best methods used for epidemiological typing of pathogens. However, the limitations of this method are the relatively high costs of implementation and the need for specialized equipment [4]. Due to this fact, different techniques based on the Polymerase Chain Reaction (PCR) are often used for determining genetic fingerprints as an alternative to PFGE. Among several PCR-based tools, the Random Amplified Polymorphic DNA-Polymerase Chain Reaction (RAPD-PCR) is a simple and cost-effective genotyping method for the differentiation of bacterial strains. In RAPD-PCR, a short primer, usually 10 nucleotides long, is applied to amplify multiple genomic loci, which are detected by an agarose gel electrophoresis as strain-specific profiles (RAPD-PCR genotypes). The major advantage of methods based on RAPD-PCR is that the knowledge of the DNA sequence of the tested microorganisms is not essential and not needed [4,5]. It should be mentioned that the development of a new RAPD-PCR method for typing of strains belonging to a particular species requires careful optimization of all components and conditions of the reaction, as any changes in the quantity of each ingredient may affect the results.

Up to date, few molecular typing methods, such as PFGE, ERIC-PCR (Enterobacterial Repetitive Intergenic Consensus-Polymerase Chain Reaction), RAPD-PCR or BOX-PCR (Polymerase Chain Reaction-mediated amplification using BOX motif primers), were used to study the genetic diversity of *T. pyogenes* strains [2,6,7,8]. However, these studies were performed only on isolates, often in limited numbers, from one animal species. It should be highlighted that a simple method developed especially for *T. pyogenes* genetic typing is not available, and there is still a lack of data about the genetic diversity among strains of different origins.

The aim of this study was to design a rapid and easy-to-perform molecular typing method based on RAPD-PCR for assessing the genetic diversity of *T. pyogenes* strains. In this investigation, *T. pyogenes* strains isolated from different animal species, including livestock and European bison (*Bison bonasus*), were studied to confirm the usefulness of the developed method.

## 2. Results

### 2.1. Optimization of RAPD-PCR

During the optimization process, from among five tested primers, the M13 primer was selected as the best for genotyping of *T. pyogenes* strains. In the reaction with this primer, the amplification yield was good, and the distribution of fragments was sufficient to allow comparatively easy interpretation of the results (Figure S1 of Supplementary Materials).

**Figure 1:**
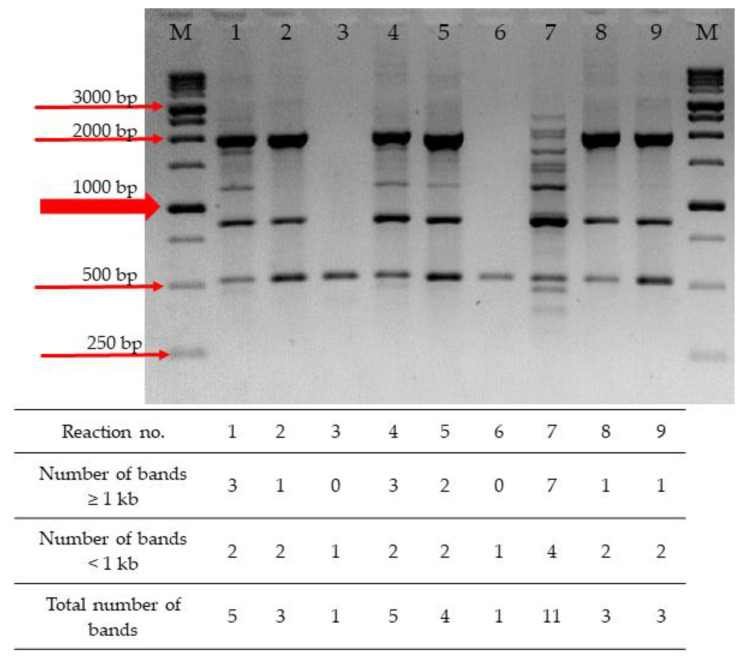
RAPD-PCR profiles obtained in the orthogonal-array reactions performed for the *T. pyogenes* 19/B strain using the M13 primer. Lane M, a molecular mass marker (1 kb DNA Ladder RTU, Nippon Genetics, Düren, Germany); lanes 1–9, the RAPD-PCR products obtained in reactions containing different concentrations of four substrates according to the orthogonal array shown in Table 1 (reactions no. 1–9, respectively).

In order to further optimize the RAPD-PCR for *T. pyogenes* differentiation, the influence of various reagent concentrations on the efficacy of the method was determined. Nine reactions, using three components with three levels each (MgCl_2_, dNTPs, a primer and a template DNA), were performed in accordance with the orthogonal array proposed by Taguchi and Wu (1980) and further Cobb and Clarkson modification (1994) [9,10]. The yield of the RAPD-PCR assay was referred to as the number of amplicons generated in each reaction. Conditions generating more DNA fragments indicate a greater yield of the assay. The concentrations of all tested reaction components affected the number and size of generated amplicons. Depending on the composition of the PCR mixture, obtained patterns comprised only from one to eleven DNA fragments (Figure 1). One of the most critical factors in RAPD-PCR seems to be the concentration of dNTPs. The most complex RAPD profile was obtained with reaction mixture no. 7, containing a low level of dNTPs (0.8 mM) and a high level of MgCl_2_ (3.5 mM) (Table 1), while the fewest amplicons were obtained in the reaction with a high level of dNTPs (a single amplicon in reactions no. 3 and 6 and three amplicons in reactions no. 2, 8, and 9).

**Figure 2:**
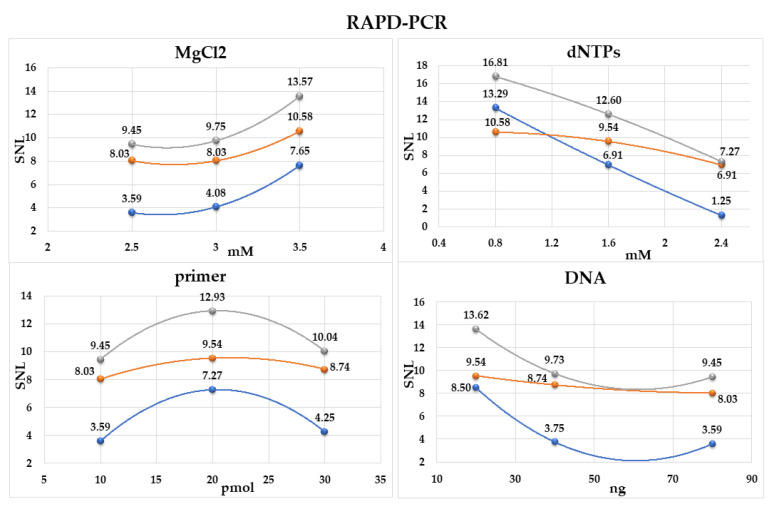
Effects of three concentrations of the tested substrates (MgCl_2_, dNTPs, primer M13, and DNA) on the SN_L_ coefficient. Grey line, total number of bands in RAPD profile; blue line, number of bands larger than 1 kb in size; orange line, number of bands less than 1 kb in size. The highest point in each curve indicates optimal conditions.

On the basis of the number of DNA fragments obtained in all experiments, we calculated the SN_L_ (signal to noise ratio) for each level of each component (Table S1). The optimal concentration was determined as the highest SN_L_ ratio among the levels (Figure 2). The concentrations of MgCl_2_ equal to 3.5 mM and of dNTPs equal to 0.8 mM were established as the most adequate. The increase in the MgCl_2_ concentration to the level of 3.5 mM and the decrease in dNTPs concentration to 0.8 mM caused a substantial increase in the SN_L_ coefficient (Figure 2). The optimal concentration of a primer was determined as 20 pmol. It was found that the optimal concentration of a DNA template should be 20 ng (Figure 2). A distinct decrease in the number of amplification products obtained in the other tested conditions of the RAPD-PCR method indicates that the use of higher or lower concentrations of the reaction components is not recommended.

### 2.2. Typing of T. pyogenes Strains by RAPD-PCR

**Figure 3:**
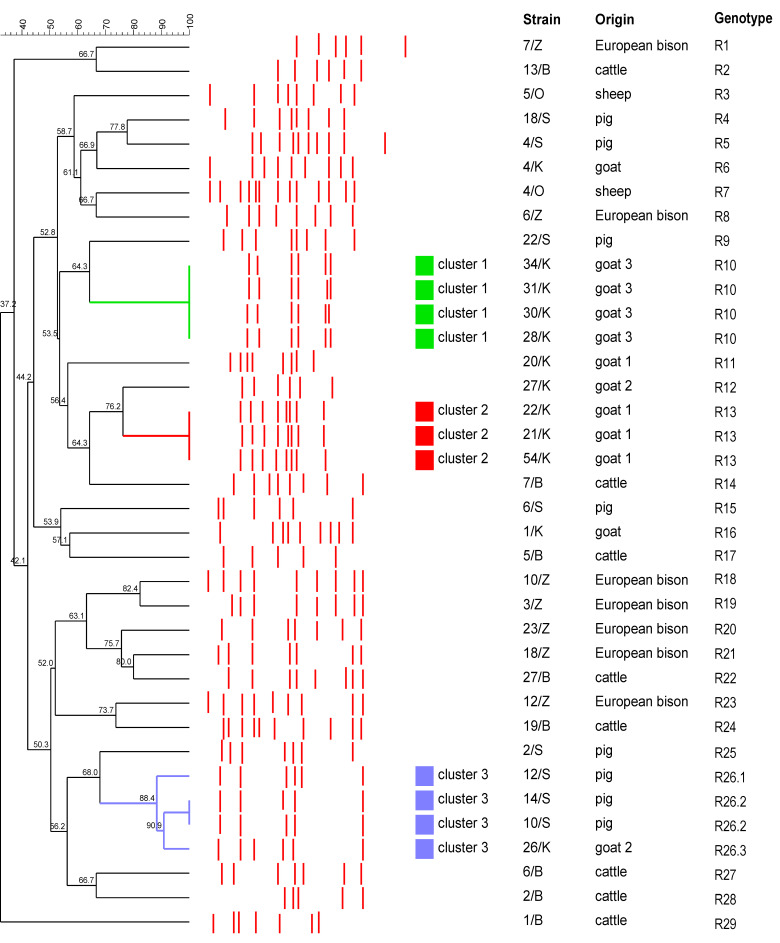
Dendrogram based on the results of 37 *T. pyogenes* strains differentiation by the developed RAPD-PCR method, illustrating a degree of similarity among tested strains. Cluster analysis was performed with BioNumerics 7.6 (Applied Maths, Sint-Martens-Latem, Belgium) and based on Dice similarity coefficient and the UPGMA analysis. Red lines shows the obtained RAPD patterns. Three clusters labeled C1 (green square), C2 (red square) and C3 (blue square) were defined from groups of closely related strains sharing at least 85% of genotype similarity. The right panel of the dendrogram shows the origin of strains and 31 distinguished genotypes.

The developed RAPD-PCR method was evaluated by typing of *T. pyogenes* strains (*n* = 37) of different origins. In the performed reactions, various profiles consisting of fragments of approximately 2520–290 bp were generated. The number of bands after the electrophoresis ranged from five to thirteen (with the average number of bands at 7.35). According to the BioNumerics analysis, thirty-one different profiles could be distinguished, and each profile was recognized as a unique RAPD-genotype (Figure 3).

Most of the studied strains (*n* = 28) represented individual genotypes (R1–R9, R11 and R12, R14–R25, R26.1 and R26.3, and R27–R29). Based on the 100% of similarity, the remaining nine strains were allocated to three genotypes (R10, R13, and R26.2). The genotype R10 was the most prevalent and was found in four strains, followed by the R13 and R26.2 genotypes included in three and two strains, respectively. All strains having the R10 genotype (strains 28/K, 30/K, 31/K, and 34/K) were isolated from the same goat (goat 3). Similarly, all strains representing the R13 genotype (strains 21/K, 22/K, and 54/K) originated from the same goat (goat 1). Two swine strains (10/S and 14/S) had the same genotype R26.2. The dendrogram based on the analysis of RAPD-PCR results obtained for the studied *T. pyogenes* strains is presented in Figure 3. Considering genetic relationships between strains with more than 85% of similarity of the RAPD genotypes, three clusters were distinguished (Figure 3). Clusters 1 and 2, each grouped the caprine strains, indistinguishable by the RAPD-PCR genotyping, isolated from the same animals. Cluster 3 included three swine strains and one caprine strain, which belonged to three separate genotypes.

The analysis of the discriminatory power of the developed RAPD-PCR method determined its high potential for investigating the genetic diversity of *T. pyogenes* strains. The value of a single numerical discriminatory index calculated for the method was 0.985 (CI95: 0.9798–0.9902) (considering all strains, also strains isolated from the same goat).

## 3. Discussion

Infections caused by *T. pyogenes* are a significant health and economic problem, especially in livestock [1,2,3]. The goal of our study was to develop a rapid and easy to perform typing method for genetic differentiation of *T. pyogenes* strains and estimation of their phylogenetic relationship, as well as for epidemiological control of infections. Up to date, several techniques have been used for molecular typing of *T. pyogenes* strains, including PFGE, MLSA, and other genomic DNA fingerprinting methods [2,6,7,8,11,12]. On the other hand, PCR used for the detection of virulence-determinant genes was frequently applied to intraspecies differentiation of these bacteria [6,11,12,13,14]. A relatively high genetic diversity within this species was shown in the PFGE analysis of a total of 180 *T. pyogenes* swine isolates [2]. However, due to the technical requirements, the time required, and costs, especially in the case of epidemiological studies involving a large number of tested samples, PFGE use is limited. Nagib et al. (2017) applied four DNA-fingerprinting techniques, ERIC-PCR, BOX-PCR, RAPD-PCR, and (GTG)5-PCR (PCR-based method using primers for short repetitive nucleotide sequences (GTG)5), for the epidemiological study of three *T. pyogenes* isolates originating from lorises [7]. However, they used the RAPD-PCR procedure (with the primer B) designed for genotyping of other bacterial species. RAPD-PCR is a random amplification of unknown genomic regions using a short (usually 10 bp) single arbitrary primer and low annealing temperature conditions, which should be optimized for typing of strains belonging to a particular species. To the best of our knowledge, this is the first work that describes the development and optimization of a RAPD-PCR procedure intended as a molecular tool for typing *T. pyogenes* strains. This technique has been demonstrated previously as a useful tool for molecular differentiation of a wide range of pathogens [15,16,17,18,19,20].

Regardless of the used method, each one should be accurately assessed in terms of its performance and applicability. The important features of good typing methods are rapidness, technical simplicity, and low cost [21]. However, several criteria are crucial for selecting a genotyping method, notably high typeability, discriminatory power, and reproducibility [21,22]. These parameters should always be assessed when a method is optimized for typing a new microorganism, as well as when any modification is made to the previously developed protocol (e.g., the use of a different primer).

In this study, typeability, discriminatory power, and reproducibility of the newly developed method, as well as its ability to differentiate *T. pyogenes* strains, were determined by the testing of 37 clinical isolates. The typeability of the method is defined as the percentage of strains from the tested population that can be assigned into a specific genotype [22]. In our study, all tested strains showed specific fingerprinting patterns, easy to interpret and compare, so the typeability of the method was 100%. 

Discriminatory power is defined as the ability of a typing method to discriminate between two unrelated isolates. This parameter considers the number of genotypes determined by the method and the relative frequency of their occurrence in the studied population [22]. The use of highly discriminatory typing methods is recommended, especially for the differentiation of closely related strains. To evaluate the discriminatory power, a single numerical index of discrimination (D) should be calculated [22]. The index is based on Simpson’s index of diversity and measures the probability that two randomly selected and unrelated strains will be assigned a different genotype. In order to obtain a precise and accurate evaluation of the D value, the isolates included in the analysis should be diverse and epidemiologically unrelated. Our study included 28 *T. pyogenes* strains that were likely to be heterogenous because they originated from various animal species (cattle, pigs, goats, sheep, and European bison) and clinical specimens, as well from different geographical regions (data not shown). The remaining nine strains were isolated from three goats (two, three, and four strains derived from each animal). The calculated D value for all tested strains (*n* = 37) was 0.985 (CI95: 0.9798–0.9902). However, if the calculation was performed only for 28 *T. pyogenes* strains of various origins, excluding the nine caprine strains that are likely to be homogenous, the D value of the developed RAPD-PCR method would be even higher (D = 0.992) (CI95: 0.9889–0.9951). Importantly, all studied *T. pyogenes* strains were successfully differentiated with the developed RAPD-PCR method, and most strains represented unique RAPD genotypes. It must be highlighted that epidemiologically related strains, derived from the same animal, had an identical RAPD genotype demonstrating their genetic identity and indicating that, in fact, they must be considered as the same strain. Interestingly, two strains, 26/K and 27/K, isolated from the same goat but from different clinical specimens (abscess and uterine swabs, respectively), belonged to two different genotypes. This finding suggests that the infection in this animal could have been caused by two clonally different or non-clonal *T. pyogenes* strains. These results confirmed that the developed method provides high typeability (100%) and discriminatory power and, consequently, is an excellent and useful tool for epidemiological investigations. Moreover, our previous study indicated that the discriminatory power of the newly developed RAPD-PCR is higher than that obtained for the ERIC-PCR typing method (unpublished data).

The third important criterion is the reproducibility of a typing method, defined as a proportion of strains in the tested population for which the same genotypes are determined in repeated testing. In our study, the results obtained in two independent replications of the RAPD-PCR assay were completely consistent, evidencing a very good reproducibility of the method.

Optimization of the typing procedure is a key step that should include the primer selection and evaluation of concentrations of crucial reaction components. The number of generated amplicons, depending on the primer-template interaction, must be sufficient to show heterogeneity between related but distinct bacterial strains. Among the five primers tested, the M13 primer was found to be the most discriminatory based on the number and size range of generated amplicons and the complexity of obtained genotypes. The primer, magnesium ions, dNTPs, and DNA template concentrations in the reaction mixture were evaluated as critical factors which determine the efficiency and specificity of the amplification. The quality of RAPD-PCR genotypes was significantly affected by both too high and too low concentrations of all tested components; thus, the optimal concentrations had to be chosen. The protocol of the optimized method provided a reliable and accurate analysis of the genetic diversity and relationship between tested *T. pyogenes* isolates.

Not only the quality but also the purity of a DNA template has a significant influence on the number and intensity of RAPD-PCR amplicons. The DNA extraction kit with column purification used in our study yielded a highly pure DNA that was stable when stored at −20 °C and allowed the reproduction of the same and readable RAPD patterns. The boiling method of extraction, although it yielded a higher concentration of DNA but of low purity, was not suitable for RAPD experiments. The RAPD patterns consisted of a smaller number of bands, smears formed, and the reproducibility of the reaction was insufficient (data not shown).

In our study, the developed RAPD-PCR fingerprinting showed a considerable genetic diversity among the tested *T. pyogenes* strains, as most strains represented distinct genotypes. Genetic events, which may determine the genomic polymorphism in bacteria, occur at frequencies dependent upon a species, a strain, and environmental conditions [21]. However, mutation and recombination rates involving the evolution of the *T. pyogenes* genome have not been estimated so far.

## 4. Materials and Methods

### 4.1. Bacterial Strains

Thirty-seven *T. pyogenes* strains isolated from various animal hosts and different suppurative infections were used in this study (12 from goats, 8 from cattle, 8 from pigs, 2 from sheep, and 7 from European bison). All strains were selected from the strain collection of the Division of Microbiology, Department of Preclinical Sciences, Institute of Veterinary Medicine, Warsaw University of Life Sciences (an origin and a source of strains are shown in Figure 3 and Table S2 of Supplementary Materials). The studied strains were isolated from individual animals from different farms, with the exception of nine strains originating from three goats (strains 28/K, 30/K, 31/K, 34/K, strains 26/K, 27/K and strains 21/K, 22/K, 54/K were isolated from the goat 3, the goat 2 and the goat 1, respectively). All strains were identified using the API Coryne test (BioMérieux, Marcy l’Etoile, France) and also by the species-specific *plo* gene PCR, as described previously [23,24]. 

Bacteria were cultured on Columbia Agar supplemented with 5% sheep blood (Graso Biotech, Starogard Gdański, Poland) at 37 °C under microaerophilic conditions for 48 h. Then, a pure culture of each strain was prepared on Tryptic Soy Broth (TSB; BioMérieux, Marcy l’Etoile, France), and after 24 h incubation, it was used for a template DNA extraction.

### 4.2. Extraction of DNA 

Genomic DNA was extracted from the 24-hour-old *T. pyogenes* culture on TSB using the GenElute^TM^ Bacterial Genomic DNA Kit (Sigma-Aldrich, Steinheim, Germany). Briefly, one milliliter of the culture was centrifuged, and a bacterial pellet was resuspended into 200 μL of the Gram-positive lysis solution supplemented with lysozyme (60 mg/mL) to enhance the effect of lysis. The sample was incubated for 30 min at 37 °C with shaking. The following isolation steps were performed according to the protocol recommended by the kit manufacturer with a minor modification of the elution step (the elution was performed using 40 µL of the Elution buffer heated to 65 °C). A DNA concentration and purity were determined with a spectrophotometer NanoDrop 1000 (Thermo Fisher Scientific, Waltham, MA, USA).

### 4.3. Optimization of RAPD-PCR Reagents

Optimization of reaction components for *T. pyogenes* RAPD-PCR was performed according to a protocol from Taguchi and Wu (1980) with Cobb and Clarkson’s modification (1994) [9,10]. The effects and interactions of four reaction substrates, MgCl_2_, dNTPs, a primer, and a DNA template, were investigated. Each component was tested at one of three concentration levels using nine reactions and according to the orthogonal array (Table 1). DNA isolated from strain 19/B was used as a template.

On the basis of the number of amplicons obtained for each concentration and taking into account the deviation between the number of amplicons and the average number of products, the SN_L_ (signal to noise ratio) was calculated according to the equation:

(1) $$SN_{L}=-10log\left(1/n\cdot \sum_{i=1}^{n}1/Y_{i}^{2}\right),$$

where *n* is the reaction number for the tested concentration levels (*n* = 3), and *Y* is the number of bands per lane increased by one (Table S1 of Supplementary Materials). The optimal concentration of each reagent was such that it gave the largest SN_L_, and its value was interfered with by polynomial regression (Figure 2).

### 4.4. RAPD-PCR Conditions

RAPD-PCR fingerprinting was performed with five primers of the following 5′–3′ sequences: primer M13, GAGGGTGGCGGTTCT [25]; Primo2, CGGCAAGGAG [26]; RP, CAGCACCCAC [26]; UBC245, CGCGTGCCAG; and UBC282, GGGAAAGCAG [27]. The optimal primer was selected based on the number and size of RAPD products obtained. All oligonucleotide primers were synthesized by Eurofins Genomics Germany GmbH (Ebersberg, Germany).

The reaction mixture, according to the results of RAPD-PCR optimization, contained 3.5 mM MgCl_2_ (Thermo Fisher Scientific, Waltham, MA, USA), 0.8 mM of each dNTP (Thermo Fisher Scientific, Waltham, MA, USA), 20 pmol of a primer, 20 ng of a DNA template, 12.5 μL DreamTaq buffer with polymerase (Thermo Fisher Scientific, Waltham, MA, USA), and water up to 25 μL. Three separate reactions for each isolate were conducted in order to confirm the reproducibility of the developed method. Following thermocycling conditions were applied: initial denaturation at 94 °C for 3 min, subsequent 40 cycles of 30 s at 94 °C, 30 s at 45 °C, and 1 min at 72 °C, followed by one final step of extension at 72 °C for 7 min. The RAPD-PCR assays were carried out in two independent replications several weeks apart

### 4.5. Gel Electrophoresis

Products of the amplification were detected by gel electrophoresis. The RAPD-PCR samples (15 µL), mixed with DNA Loading Dye (6× Thermo Fisher Scientific, Waltham, MA, USA), were separated on 2.0% agarose gel stained with Midori Green Advance DNA Stain (Nippon Genetics, Düren, Germany), in Tris-borate-EDTA buffer for 2.5 h at 90 mA. The 1kb DNA Ladder (Nippon Genetics, Düren, Germany) was used as a DNA size marker. The results were visualized, photographed, and analyzed using the Gel Doc^TM^ EZ Imager and Image Lab software, ver. 5.2.1 (Bio-Rad, Hercules, CA, USA). 

### 4.6. Analysis of the Amplification Results

The RAPD-PCR results were analyzed by BioNumerics software version 7.6 (Applied Maths, Sint-Martens-Latem, Belgium), and a cluster analysis was performed by Unweighted Pair Group Method with Arithmetic Mean (UPGMA) using Dice similarity coefficient with optimization and position tolerance set at 1%. Strains were clustered using an 85% homology cut-off, above which strains were considered to be closely related and assigned to the same cluster.

### 4.7. Calculation of a Discriminatory Index

In order to assess the discriminatory power of the developed RAPD-PCR method for genotyping of *T. pyogenes*, a single numerical index of discrimination (D) described by Hunter and Gaston (1988) was used [22]. The index is based on the probability that two independent isolates, sampled randomly from the tested population, are classified into different genotypes. Genotypes were established based on 100% similarity of obtained patterns.

## 5. Conclusions

The results of our study demonstrated that the developed RAPD-PCR method has a high typeability and discriminatory power, as shown by the high index D value, and thus provides a useful and practical means of genotyping *T. pyogenes* strains. It also has the advantages of rapidity, technical simplicity, and easy fingerprint interpretation. Consequently, the developed method can be an excellent first-line molecular typing tool, especially when an epidemiological analysis requires investigations of a large number of *T. pyogenes* isolates.

## Figures and Tables

**Table 1:** The orthogonal array for optimizing RAPD-PCR by testing four reaction components, each at three different concentration levels.

Reaction No.	MgCl_2_ (mM)	dNTPs (mM)	Primer (pmol)	DNA (ng)
1.	2.5	0.8	10	20
2.	2.5	1.6	20	40
3.	2.5	2.4	30	80
4.	3.0	0.8	20	80
5.	3.0	1.6	30	20
6.	3.0	2.4	10	40
7.	3.5	0.8	30	40
8.	3.5	1.6	10	80
9.	3.5	2.4	20	20

## Data Availability

The data presented in this study are available on request from the corresponding authors.

## Supplementary Materials

The following supporting information can be downloaded at: https://www.mdpi.com/article/10.3390/pathogens11050562/s1, Figure S1: RAPD-PCR profiles obtained with different random primers, RP, M13, Primo2, UBC245, or UBC282 for strain 19/B, 22/K, 6/S, and 6/Z; Table S1: The values of SN_L_ coefficient obtained for various concentrations of the tested RAPD-PCR components. Table S2: Characteristics of the *T. pyogenes* strains included in this study.
