# Supplementary material for: RAPD-PCR-Based Fingerprinting Method as a Tool for Epidemiological Analysis of Trueperella pyogenes Infections
Source: Pathogens. 2022 May 10;11(5):562. doi: 10.3390/pathogens11050562 (PMC9147813; doi:10.3390/pathogens11050562)
Supplement: Supplementary file 1 [file pathogens-11-00562-s001.zip › Table S1.pdf]

Table S1. The values of SN_L coefficient obtained for various concentrations of the tested RAPD-PCR components.

Component concentration		Number of bands ≥1 kb*	SN_L	Number of bands <1 kb*	SN_L	Total number of bands*	SN_L
**$MgCl_2$ (mM)**	**2.5**	4…2…1	3.59	3…3…2	8.03	6…4…2	9.45
	**3.0**	4…3…1	4.08	3…3…2	8.03	6…5…2	9.75
	**3.5**	8…2…2	7.65	5…3…3	10.58	12…4…4	13.57
**dNTPs (mM)**	**0.8**	4…4…8	13.29	3…3…5	10.58	6…6…12	16.81
	**1.6**	2…3…2	6.91	3…3…3	9.54	4…5…4	12.60
	**2.4**	1…1…2	1.25	2…2…3	6.91	2…2…4	7.27
**Primer M13 (pmol)**	**10**	4…1…2	3.59	3…2…3	8.03	6…2…4	9.45
	**20**	2…4…2	7.27	3…3…3	9.54	4…6…4	12.93
	**30**	1…3…8	4.25	2…3…5	8.74	2…5…12	10.04
**DNA (ng)**	**20**	4…3…2	8.50	3…3…3	9.54	6…5…4	13.62
	**40**	2…1…8	3.75	3…2…5	8.74	4…2…12	9.73
	**80**	1…4…2	3.59	2…3…3	8.03	2…6…4	9.45

* Number of bands obtained in each of the three reactions were increased by one and these values were used directly to determine the SN_L coefficient (*Y*).
