# Supplementary material for: RAPD-PCR-Based Fingerprinting Method as a Tool for Epidemiological Analysis of Trueperella pyogenes Infections
Source: Pathogens. 2022 May 10;11(5):562. doi: 10.3390/pathogens11050562 (PMC9147813; doi:10.3390/pathogens11050562)
Supplement: Supplementary file 1 [file pathogens-11-00562-s001.zip › Table S2.pdf]

Table S2. Characteristic of the *T. pyogenes* strains included in this study.

No.	Strain	Host	Source	Year of isolation	Geographical origin
**1.**	1/B	cattle	mastitis	2015	Kuyavian-Pomeranian
**2.**	2/B	cattle	mastitis	2015	Kuyavian-Pomeranian
**3.**	5/B	cattle	lung abscess	2015	Kuyavian-Pomeranian
**4.**	6/B	cattle	abscess	2015	Kuyavian-Pomeranian
**5.**	7/B	cattle	metritis	2015	Kuyavian-Pomeranian
**6.**	13/B	cattle	metritis	2015	Kuyavian-Pomeranian
**7.**	19/B	cattle	mastitis	2015	Kuyavian-Pomeranian
**8.**	27/B	cattle	mastitis	2015	Kuyavian-Pomeranian
**9.**	3/Z	European bison	balanoposthitis	2015-16	Bialowieza National Park
**10.**	6/Z	European bison	balanoposthitis	2015-16	Bialowieza National Park
**11.**	7/Z	European bison	balanoposthitis	2015-16	Bialowieza National Park
**12.**	10/Z	European bison	abscess	2015-16	Bialowieza National Park
**13.**	12/Z	European bison	abscess	2015-16	Bialowieza National Park
**14.**	18/Z	European bison	balanoposthitis	2015-16	Bialowieza National Park
**15.**	23/Z	European bison	pneumonia	2015-16	Bialowieza National Park
**16.**	2/S	pig	pneumonia	2015	Lublin Voivodeship
**17.**	4/S	pig	pneumonia	2015	Lublin Voivodeship
**18.**	6/S	pig	heart abscess	2015	Lublin Voivodeship
**19.**	10/S	pig	pneumonia	2015	Lublin Voivodeship
**20.**	12/S	pig	liver abscess	2015	Lublin Voivodeship
**21.**	14/S	pig	join abscess	2015	Lublin Voivodeship
**22.**	18/S	pig	pneumonia	2015	Lublin Voivodeship
**23.**	22/S	pig	brain abscess	2015	Lublin Voivodeship
**24.**	1/K	goat	abscess	2010	Greater Poland Voivodeship
**25.**	4/K	goat	abscess	2012	Greater Poland Voivodeship
**26.**	20/K	goat 1	lung abscess	2015	Greater Poland Voivodeship
**27.**	21/K	goat 1	mammary gland	2015	Greater Poland Voivodeship
**28.**	22/K	goat 1	lymph node	2015	Greater Poland Voivodeship
**29.**	54/K	goat 1	lymph node	2015	Greater Poland Voivodeship
**30.**	26/K	goat 2	vagina abscess	2016	Greater Poland Voivodeship
**31.**	27/K	goat 2	lung abscess	2016	Greater Poland Voivodeship
**32.**	28/K	goat 3	lung abscess	2016	Greater Poland Voivodeship
**33.**	30/K	goat 3	lung abscess	2016	Greater Poland Voivodeship
**34.**	31/K	goat 3	bronchial abscess	2016	Greater Poland Voivodeship
**35.**	34/K	goat 3	lung abscess	2016	Greater Poland Voivodeship
**36.**	4/O	sheep	abscess	2007	Greater Poland Voivodeship
**37.**	5/O	sheep	Ropień kości	2007	Greater Poland Voivodeship
