# Supplementary figures and images for: RAPD-PCR-Based Fingerprinting Method as a Tool for Epidemiological Analysis of Trueperella pyogenes Infections
Source: Pathogens. 2022 May 10;11(5):562. doi: 10.3390/pathogens11050562 (PMC9147813; doi:10.3390/pathogens11050562)

### Primer RP

M 19/B 22/K 6/S 6/Z

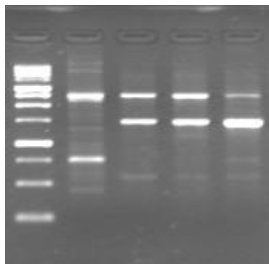

### Primer M13

M 19/B 22/K 6/S 6/Z

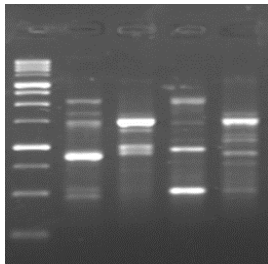

### Primer Primo2

M 19/B 22/K 6/S 6/Z

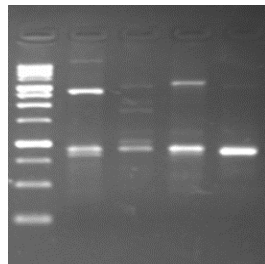

### Primer UBC245

M 19/B 22/K 6/S 6/Z

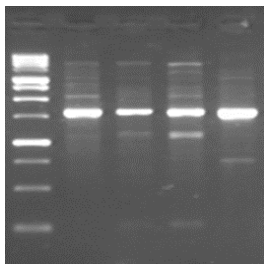

### Primer UBC282

M 19/B 22/K 6/S 6/Z

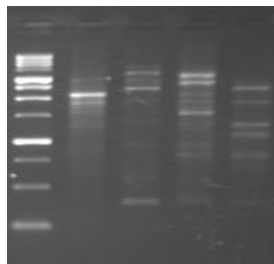

Supplement: Supplementary file 1 [file pathogens-11-00562-s001.zip › Figure S1.pdf]
